# Copper doped ceria porous nanostructures towards a highly efficient bifunctional catalyst for carbon monoxide and nitric oxide elimination†
†Electronic supplementary information (ESI) available: details of the experiments; XRD, SEM, TGA and EDX patterns of the products; the CO catalytic activity of CeO_2_:Cu^2+^ porous nanomaterials. See DOI: 10.1039/c5sc00129c



**DOI:** 10.1039/c5sc00129c

**Published:** 2015-02-10

**Authors:** Shanlong Li, Nengli Wang, Yonghai Yue, Guangsheng Wang, Zhao Zu, Yu Zhang

**Affiliations:** a Key Laboratory of Bio-Inspired Smart Interfacial Science and Technology of Ministry of Education , School of Chemistry and Environment , Beihang University , Beijing , 100191 , P. R. China . Email: jade@buaa.edu.cn; b International Research Institute for Multidisciplinary Science , Beihang University , Beijing , 100191 , P. R. China; c School of Material Science and Engineering , Changchun University of Science and Technology , Changchun , 130022 , P. R. China

## Abstract

Copper doped ceria porous nanostructures with a tunable BET surface area were prepared using an efficient and general metal–organic-framework-driven, self-template route. The XRD, SEM and TEM results indicate that Cu^2+^ was successfully substituted into the CeO_2_ lattice and well dispersed in the CeO_2_:Cu^2+^ nanocrystals. The CeO_2_:Cu^2+^ nanocrystals exhibit a superior bifunctional catalytic performance for CO oxidation and selective catalytic reduction of NO. Interestingly, CO oxidation reactivity over the CeO_2_:Cu^2+^ nanocrystals was found to be dependent on the Cu^2+^ dopants and BET surface area. By tuning the content of Cu^2+^ and BET surface area through choosing different organic ligands, the 100% conversion temperature of CO over CeO_2_:Cu^2+^ nanocrystals obtained from thermolysis of CeCu–BPDC nanocrystals can be decreased to 110 °C. The porous nanomaterials show a high CO conversion rate without any loss in activity even after five cycles. Furthermore, the activity of the catalysts for NO reduction increased with the increase of BET surface, which is in accordance with the results of CO oxidation.

## Abstract 2

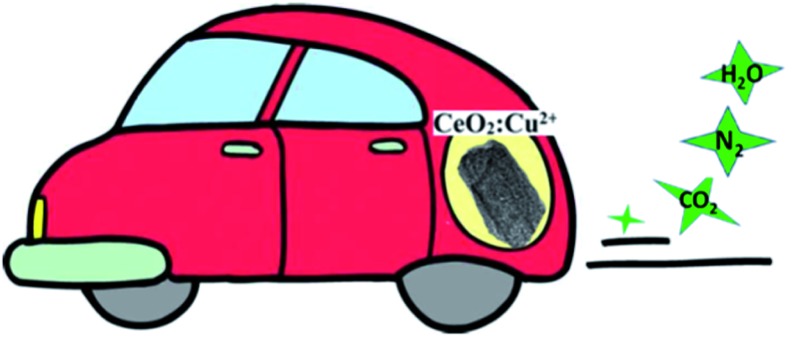
Cu^2+^ doped CeO_2_ porous nanomaterials were synthesized by calcining CeCu–MOF nanocrystals. They exhibited a superior bifunctional catalytic performance for CO oxidation and selective catalytic reduction of NO.

## Introduction

Carbon monoxide (CO) and nitric oxides (NO_*x*_) are hazardous pollutants emitted from the combustion of fossil fuels in coal fired power stations and in automobiles.^1–3^ Consequently, they have seriously negative impacts on human health in terms of asthma, emphysema, bronchitis, as well as heart diseases. In response to this, much research attention has been focused on the development of novel and highly efficient catalysts for CO oxidation and NO_*x*_ reduction.^4–10^ With a high abundance, cerium oxidation (CeO_2_) has always been introduced to three-way catalysts for the elimination of engine exhaust pollutants, due to the abundant oxygen vacancy defects, high oxygen storage capacity, and relatively easy shuttles between the III and IV oxidation states, which give rise to enhanced rates of the oxidation reaction.^11–14^ Up until now, intensive efforts have been devoted to CeO_2_ supported noble metals such as Ag, Pt, Pd, Ru and Ir, which have remarkably more active and long-term stability as catalysts at a relatively low temperature for CO oxidation reaction and selective catalytic reduction of NO (SCR). Unfortunately, they suffer from serious drawbacks including high costs, limited availability and relatively low selectivity at high temperatures, which limits their widespread application.^15–17^ On the other hand, considering the limited availability of noble metals, more attention has also been focused on the development of non-precious catalysts including CuO, Fe_3_O_4_, Co_3_O_4_, MnO_2_, MoO_3_ and NiO.^18^ Among them, copper-ceria catalysts are gaining tremendous attention and have been found to be the most interesting systems because of their higher reducibility correlated with the synergetic effect between copper and ceria at low temperatures.^19,20^ They are also low-cost, environmentally friendly, and chemically and thermodynamically stable metal oxides. Furthermore, copper-ceria catalysts exhibit significant bifunctional catalytic removal properties for NO with CO originating from stationary and mobile sources, which is one of the most important reactions occurring in automotive catalytic converters in recent years due to its importance for environmental protection.^21,22^

Porous micro- and nano-structures with controllable size, shape, composition, and interior architecture have attracted great interest because of their promising applications in various areas including catalysis, drug delivery, gas sensors, energy conversion/storage systems, *etc.* ^23–30^ A great number of novel approaches based on different mechanisms, such as the Kirkendall effect, galvanic replacement, chemical etching, thermal decomposition, and self-templating have been developed to prepare materials holding various porous structures.^31–34^ On the other hand, metal–organic frameworks (MOFs) are a big class of hybrid materials that have attracted considerable attention due to their intriguing structural motifs and various potential applications.^35–40^ Recently, several strategies including traditional solvothermal syntheses and microwave-assisted methods have been developed to prepare nanosized MOF crystals with designed morphology.^41–43^ More importantly, nanosized MOF crystals can be easily converted to nanoporous metal oxides with highly nanocrystalline frameworks and can maintain their original morphology by a self-sacrificed templated route *via* thermolysis under a certain atmosphere.^44,45^ The sacrificial templates could directly determine the shape and size of the derived porous structures by engaging themselves as the consumable reactants for shell construction. It should be noted that, referring to MOF nanocrystals as sacrificial templates, not only the shape and size can be well controlled, but also the surface area can be easily tuned by choosing different organic ligands.

In previous work, they have successfully proposed an easily scalable method for the synthesis of mono- or bi-metallic nanosized Ln–MOFs crystals.^43,46–51^ This method was used in this work to produce Ce(Cu)–BTC nanosized crystals (BTC = 1,3,5-benzenetricarboxylic acid). Furthermore, an *in situ* thermolysis approach is then introduced to obtain a novel kind of porous nanostructure composed of Cu^2+^ highly dispersed CeO_2_ materials. The method is surfactant-free and scalable at a low cost. Unexpectedly, the as-prepared CeO_2_:Cu^2+^ porous nanocrystals show an excellent bifunctional catalytic performance for CO oxidation and the selective catalytic reduction of NO.

## Results and discussion

The chemical composition and crystal structure of the samples were determined by X-ray powder diffraction (XRD). As shown in Fig. S1,† the XRD pattern of the Ce(BTC)(H_2_O)_6_ nanocrystals agreed well with that reported in the literature,^41^ which suggests high phase purity and integrity of the MOF structure. Moreover, the XRD patterns of the Cu^2+^ doped (10% content with Ce) Ce(BTC)(H_2_O)_6_ nanostructures did not show any additional peaks which means that the additional Cu^2+^ does not affect the crystal structure of Ce(BTC)(H_2_O)_6_ (Fig. S1†). The morphology of the Ce–MOF nanocrystals was determined using field emission scanning electron microscopy (FE-SEM) as shown in Fig. S2.† Interestingly, the Cu^2+^ cation doped samples retain the original morphology and no other impurities were observed (Fig. S2†).

**Fig. 1:**
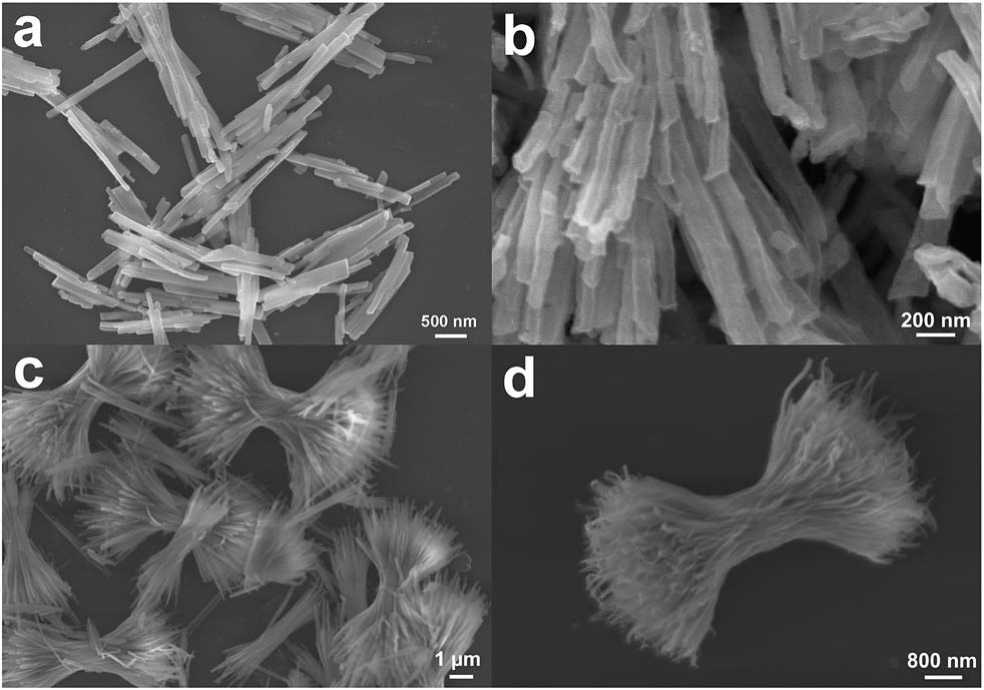
SEM images of porous CeO_2_:Cu^2+^ nanorods and nanobundles.

**Fig. 2:**
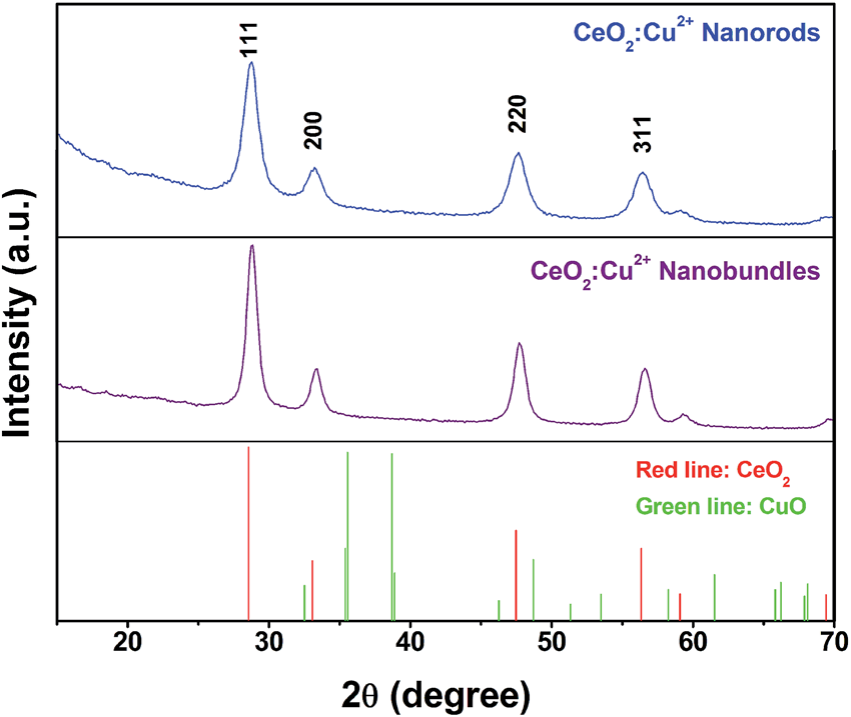
XRD patterns of porous CeO_2_:Cu^2+^ nanorods and nanobundles.

For CeCu(BTC)(H_2_O)_6_ nanocrystals prepared under high concentrations, the sample shows a morphology of rod-shaped nanocrystals with typical diameters in the range of 50–100 nm and lengths of 1–5 μm (Fig. S2a†). For the CeCu(BTC)(H_2_O)_6_ nanocrystals prepared under low concentrations, uniform 3D superstructures composed of abundant nanorods were observed with a mean length of about 5 μm (Fig. S2b†). These nanorods are well-aligned and have a smooth surface, with a diameter of about 50–100 nm and lengths of 1–5 μm. No other impurities are observed, which means that the Cu^2+^ are successfully well dispersed into the Ce(BTC)(H_2_O)_6_ nanocrystals and this was confirmed well with the XRD results. After heating the Ce(Cu)–BTC MOF nanocrystals at 600 °C in air for 3 h, nanoporous crystalline CeO_2_:Cu^2+^ nanocrystals without altering the original morphology can be obtained, and many holes formed in the nanocrystals due to removing the organic part of the MOF crystals (Fig. 1). Thermogravimetric analysis shows that a total of 64.5% weight loss was observed from ambient temperature to 600 °C (Fig. S3†), which is consistent with the calculated value of 64.0% considering the loss of the organic portion. The XRD patterns of the heat treated samples (Fig. 2) illustrated that all the diffraction peaks can be indexed to a CeO_2_ phase (JCPDS 79-0825). No crystalline phase ascribed to the CuO or Cu species can be observed, indicating that Cu^2+^ has been successfully substituted into the CeO_2_ lattice. The only difference was that the peaks of the Cu^2+^ doped sample are slightly right shifted about 0.2° compared with those of the pure CeO_2_ phase (Fig. S4†). The right shift might be caused by the smaller ionic radius of Cu^2+^ (0.72 Å) than that of Ce^4+^ (0.97 Å). Therefore, the existence of only a cubic fluorite phase in the XRD patterns and the right shift of the diffraction peaks indicates that the copper species have been partly incorporated into the ceria lattice and formed a Cu–Ce–O solid solution. This phenomenon was previously reported by Yang *et al.* and other researchers.^52–54^ In their study, the Cu^2+^ cations were not only stabilized at the O-hollow site, but also incorporated into the CeO_2_ lattice. To confirm the chemical composition of the synthesized products, energy dispersive X-ray spectroscopy (EDX) was then performed. The representative EDX patterns recorded from the CeO_2_:Cu^2+^ (Fig. S5†) revealed that the samples are composed of Ce, Cu, and O elements, and the ratio of the Ce and Cu elements in different morphology products is calculated to be close to a theoretical ratio of 9 : 1.

**Fig. 3:**
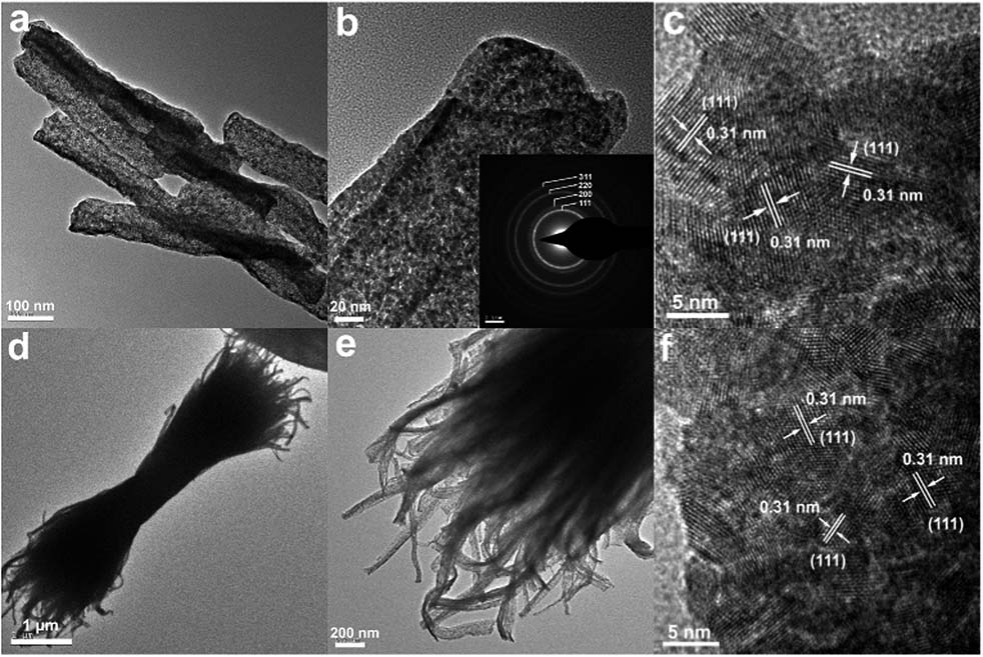
TEM (a, b, d and e), HRTEM (c and f) and SAED (inset b) images of the porous CeO_2_:Cu^2+^ nanorods and nanobundles.

**Fig. 4:**
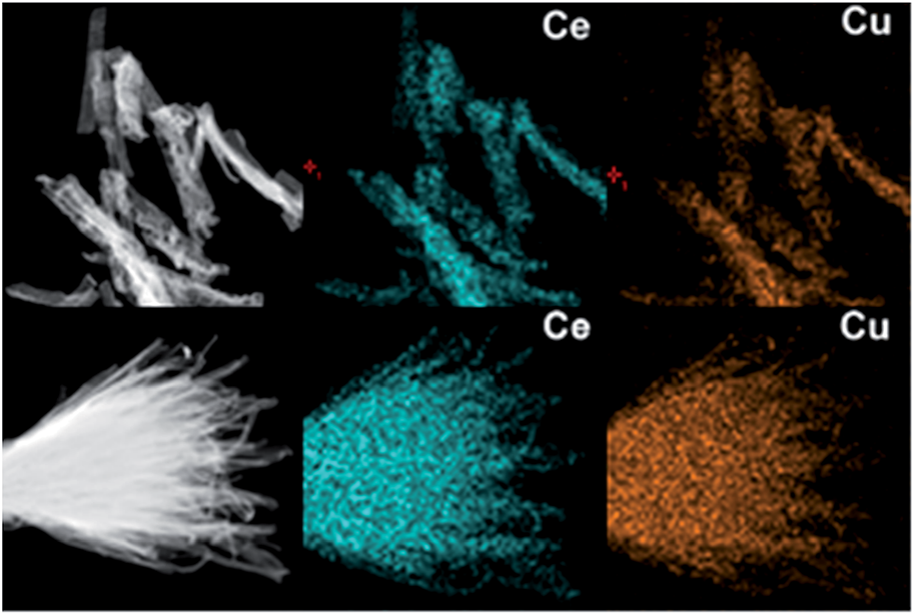
The mapping analysis of porous CeO_2_:Cu^2+^ nanorods and nanobundles.

The transmission electron microscopy (TEM) images in Fig. 3 show that the as-obtained CeO_2_:Cu^2+^ nanomaterials are porous and do not expand during the thermal treatment process. A single porous nanorod is depicted in Fig. 3b. It can be easily found that each CeO_2_:Cu^2+^ nanorod is made up of numerous nanoparticles with particle sizes ranging from 5 to 10 nm. The interior void space can be clearly observed. Furthermore, the outstanding interfacial contact can be envisaged due to the tight connection within each CeO_2_:Cu^2+^ nanoparticle (Fig. 3b). The size of the individual nanoparticles is consistent with the dimensions calculated by the Scherrer equation from their XRD patterns (Fig. 1). It can be seen from the high resolution TEM images of the specific parts of the dispersed nanorod (Fig. 3c), that the lattice spacing (0.31 nm) corresponds well with the characteristic (111) planes of the fluorite CeO_2_ phase. The nanocrystallinity is also confirmed by the presence of rings in the selected area electron diffraction (SAED) as shown in the inset of Fig. 3b. The SAED ring pattern corresponds well to the fluorite structure of CeO_2_. The TEM results of the calcined nanobundles are similar with the CeO_2_:Cu^2+^ porous nanorods (Fig. 3d–f). The mapping analysis (Fig. 4) of the hybrid nanostructure also identified the composition, in which Ce and Cu elements homogeneously spread inside the nanocrystals, which further confirm the fact that the Cu^2+^ cations are well dispersed into the porous CeO_2_ nanocrystals.

**Fig. 5:**
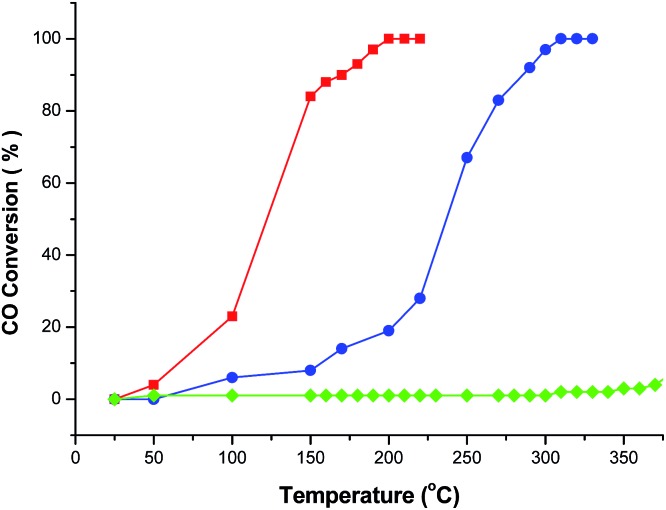
CO conversion *vs.* reaction temperature over the different catalysts (red line: CeO_2_:Cu^2+^ nanorods, blue line: CeO_2_:Cu^2+^ nanobundles, and green line: pure CeO_2_ porous nanorods).

**Fig. 6:**
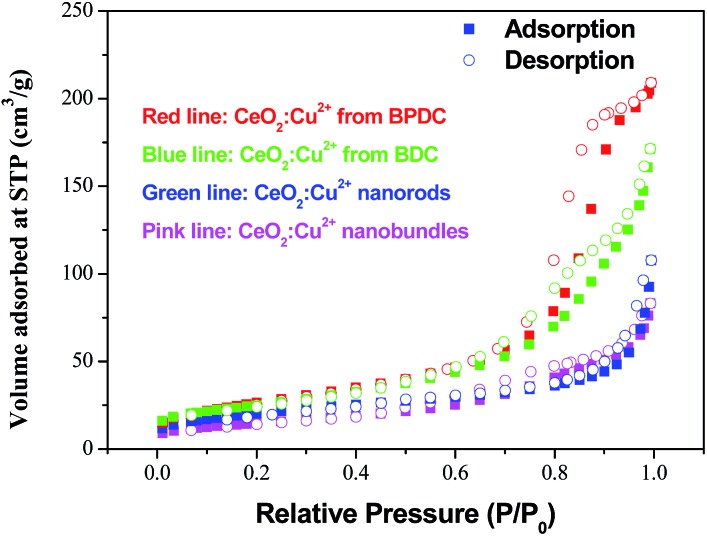
N_2_ adsorption–desorption isotherm of the CeO_2_:Cu^2+^ porous nanocrystals.

Cu^2+^ doped CeO_2_ materials are generally considered to be the most important and prevalent catalysts in the fields of exhaust emission control and fuel cells. In this context, CO catalytic oxidation was then carried out to evaluate the catalytic performance of the obtained products. For comparison, pristine CeO_2_ porous nanomaterials prepared by calcining the original Ce–BTC at 600 °C for 3 h were also employed for the CO catalytic oxidation. As shown in Fig. 5, it was found that for the conversion of CO into CO_2_, the 100% conversion temperature of the four samples increase as follows: CeO_2_:Cu^2+^ nanorods (190 °C) < CeO_2_:Cu^2+^ nanobundles (290 °C) < pure CeO_2_ nanorods (>300 °C). This means that the CeO_2_:Cu^2+^ nanorods exhibited much higher activity than both the CeO_2_:Cu^2+^ nanobundles and the pristine porous CeO_2_ nanorods, which might be attributed to the homogeneously dispersed Cu^2+^ and a good interfacial contact within each CeO_2_:Cu^2+^ nanoparticle formed legitimately after calcining the original Ce(Cu)–BTC nanocrystals at certain temperature. In the initial experimental process, Cu^2+^ and Ce^3+^ were mixed together with BTC, thus numerous Ce–O–Cu linkages could be formed to build up a complex structure during the formation of Ce(Cu)–BTC MOF. In addition, adequate Ce–O–Cu linkages may also be formed at the interface within the CeO_2_:Cu^2+^ nanoparticles, leading to significantly enhanced interfacial interactions. The many Ce–O–Cu linkages and good interfacial contact result in much easily adsorbed CO. As a result, the CO catalytic activity of the CeO_2_:Cu^2+^ porous nanomaterials is greatly enhanced compared to that of the pristine CeO_2_ porous nanorods. Additionally, the CeO_2_:Cu^2+^ catalyst was recovered after the catalytic reaction to further characterize its stability. Interestingly, the XRD pattern and SEM image (Fig. S6†) demonstrate that both the crystal phase and the porous structure of the CeO_2_:Cu^2+^ catalysts are well preserved after the catalytic reaction, demonstrating the superior stability of the as-produced composite catalysts. Furthermore, the catalytic properties of the CeO_2_:Cu^2+^ nanorods and CeO_2_:Cu^2+^ nanobundles are normalized by the Brunauer–Emmett–Teller (BET) surface area. N_2_ adsorption–desorption isotherms of the CeO_2_:Cu^2+^ nanorods and CeO_2_:Cu^2+^ nanobundles are displayed in Fig. 6, which suggest specific surface areas of 72.12 and 52.90 m^2^ g^–1^, respectively. The main reason for the superior catalytic activity of the CeO_2_:Cu^2+^ porous nanorods than that of the CeO_2_:Cu^2+^ porous nanobundles can thus be attributed to the high BET surface – high BET surface means high CO absorption and CO conversion rate of the CeO_2_:Cu^2+^ porous nanorods.

Based upon the above results and the mechanisms of Cu–Ce catalysts suggested in the literature, a potential reaction pathway for CO oxidation over CeO_2_:Cu^2+^ porous nanocrystals is proposed. The mechanism of the reaction is linked to the adsorption and desorption of the produced molecules during the reaction on the nanocatalyst surface. As discussed above, numerous Cu–Ce–O linkages may be formed in the porous CeO_2_:Cu^2+^ nanocrystals during the thermolysis of the Ce(Cu)–BTC MOF nanostructures. As a result, the enormous Cu–Ce–O linkages and good interfacial contact facilitate the adsorption of CO. It is well accepted that CeO_2_ is rich in oxygen vacancy defects and holds a large oxygen storage capacity, which is beneficial for the CO catalytic oxidation. Furthermore, the porous structures induced high surface could provide more active sites to improve the catalytic activity. Interestingly, the Cu^2+^ content also plays an important role in enhancing the catalytic activity. Controlled experiments were carried out to compare the catalytic activity within different Cu^2+^ concentrations. As shown in Fig. S7,† it can be found that the sample with 10% Cu^2+^ shows the highest catalytic activity (Fig. S7†). It is well understood that reducing Cu^2+^ content causes less CO adsorption. However, by increasing the Cu^2+^ content, although the active sites increased, the agglomeration of particles resulted in decreasing activity. In particular, when the doped concentration is 20%, the tolerance of the CeO_2_ crystal lattices was broken and CuO emerged (Fig. S8†). Reusability is also an important parameter for a catalytic material.

We selected CeO_2_:Cu^2+^ nanorods as the model catalyst and tested the reusability of it by repeated heat treatment in 1% CO gas. After five successive cycles, the CeO_2_:Cu^2+^ nanorods could still maintain a 100% conversion rate of CO into CO_2_. This result clearly shows that the as-obtained CeO_2_:Cu^2+^ nanorod catalyst is stable and active under long-term high-temperature catalytic conditions (Fig. S9†).

**Fig. 7:**
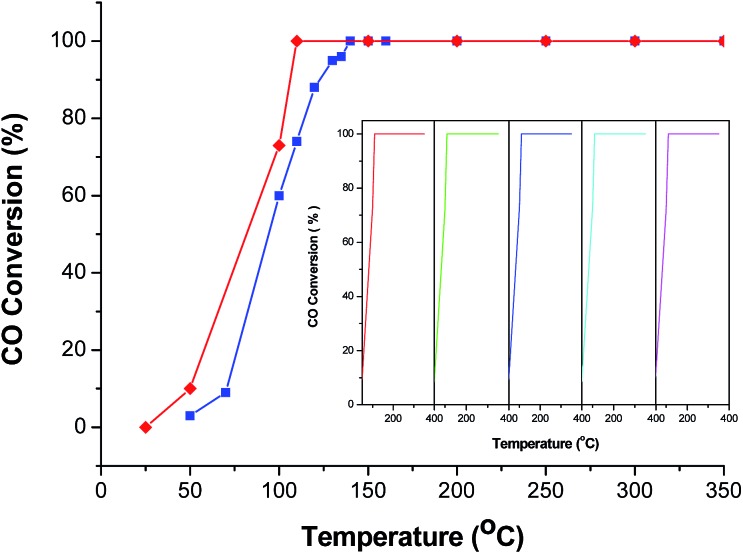
CO conversion *vs.* reaction temperature over the different catalysts (red line: CeO_2_:Cu^2+^ nanocrystals obtained from Ce(Cu)–BPDC and blue line: CeO_2_:Cu^2+^ nanocrystals obtained from Ce(Cu)–BDC. Inset is the catalytic cycles of the CeO_2_:Cu^2+^ nanocrystals obtained from Ce(Cu)–BPDC.

In order to further improve the CO catalytic activity, additional works have been performed. As it is well-known, MOFs with infinite one-, two-, or three-dimensional structures are assembled with metal ions or polynuclear clusters as nodes and organic ligands as linkers. Therefore, it is convenient to predict the final structures of the desired crystalline products since many factors such as metal ions, organic ligands, solvent systems, pH and temperature may have a great influence on the self-assembly process. As discussed above, the catalytic properties of the CeO_2_:Cu^2+^ nanomaterials are increased with an increase of BET surface area. Inspired by this idea and previous studies, different organic ligands, namely, benzenedicarboxylic acid (BDC) and 4,4′-biphenyldicarboxylic acid (BPDC), were introduced to the reaction. In the obtained Ce(Cu)–BDC and Ce(Cu)–BPDC nanocrystals, the content of the organic part is slightly different. After thermolysis, the Ce–MOFs nanocrystals convert into porous CeO_2_:Cu^2+^ products with a different BET surface area. The morphology of the products is shown in Fig. S10.† From the N_2_ adsorption–desorption isotherms of the CeO_2_:Cu^2+^ nanomaterials (Fig. 6), it can be calculated that the specific surface areas are 88.11 (Ce(Cu)–BDC), and 97.00 (Ce(Cu)–BPDC) m^2^ g^–1^, respectively. The catalytic results indicate that, for the conversion of CO into CO_2_, the 100% conversion temperature of the two samples are 110 and 140 °C for Ce(Cu)–BPDC and Ce(Cu)–BDC nanocrystals, respectively (Fig. 7). This means that the samples with a higher BET surface area exhibited a much higher activity, which is in good agreement with the above results. Moreover, in the recycling experiments, it was obvious that after five cycles the sample still maintained a high CO conversion rate without any loss of activity, as shown in Fig. 7 (inset).

**Fig. 8:**
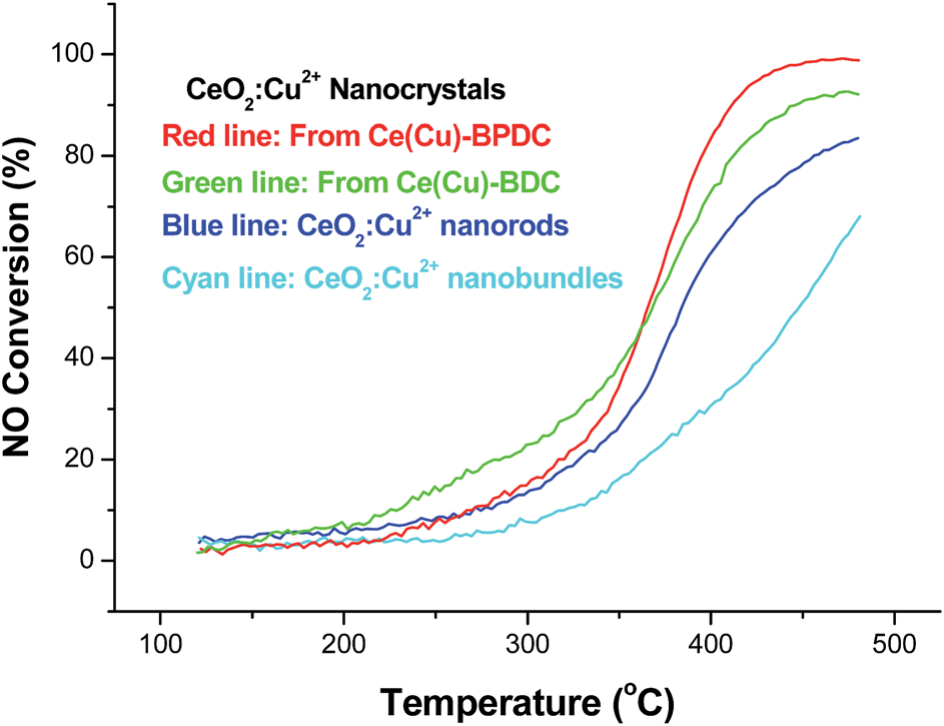
Profiles of NO conversion as a function of reaction temperature over the different CeO_2_:Cu^2+^ nanocrystals.

To further explore the catalytic activity of the obtained catalyst, NO reduction over the porous nanocrystals was then investigated. The conversion of NO and CO related to reaction temperature over CeO_2_:Cu^2+^ in the NO/CO test as shown in Fig. 8. It is found that all the prepared catalysts were active from 120 °C onwards, and that the conversion of NO is even less than 20% below 300 °C. Over the CeO_2_:Cu^2+^ nanocrystals obtained from Ce(Cu)–BPDC, NO and CO conversion reached *ca.* 90% at 400 °C. The conversion is lower than the NO conversion reported for selective catalytic reduction (SCR) with strong reducing agents, such as SCR with NH_3_ or SCR with C_3_H_6_. At high temperatures (above 380 °C), the CeO_2_:Cu^2+^ nanocrystals showed increased and sustained activity to convert up to 60% of the NO. At temperatures of 400–450 °C, the rate of conversion of NO by CO observed is comparable to some reports of SCR by NH_3_ over copper based catalysts. Although the temperature of 100% conversion of NO is still high compared with other catalysts, the catalytic process is free of acrid, corrosive, and/or organic gases. Thus this kind of catalyst also maintains great superiority in post-treatment and reaction-equipment. For each catalyst and all the reaction conditions, there was no NO_2_ recorded by the chemiluminescence NO_*x*_ analyzer, which confirms the selective reduction of NO to N_2_. Therefore, the reduction of NO in the presence of CO is believed to proceed in two steps, first a partial reduction of NO to give N_2_O, and then a subsequent reduction of N_2_O to N_2_. The activity of the catalysts for NO reduction increased with the increase in BET surface, which is according to the results of CO oxidation. From the different NO conversion curve of the catalysts, the results demonstrated that the CeO_2_:Cu^2+^ nanocrystals obtained from Ce(Cu)–BPDC are superior to other nanocrystals in terms of catalytic activity.

## Conclusions

In this report, we show a rapid, efficient and general method for the synthesis of Cu^2+^ doped CeO_2_ porous nanomaterials by calcining Ce(Cu)–MOF nanocrystals under a high temperature. Interestingly, the Cu^2+^ can be well substituted into the CeO_2_ lattice due to the formation of Cu–Ce–O linkages in the Ce(Cu)–MOF nanocrystals. The content of the Cu^2+^ and BET surface area can be easily tuned through the amount of reactant and organic ligand. When employed as the catalyst in the CO oxidation reaction, this novel material exhibits an excellent catalytic activity with 100% conversion at low temperature and exhibits a dependence on the Cu^2+^ dopants and BET surface area. Furthermore, CeO_2_:Cu^2+^ nanocrystals also exhibit an excellent catalytic performance of selective catalytic reduction of NO. The stability and activity of the CeO_2_:Cu^2+^ nanocatalyst meet the key requirements for their application in catalysis. We envisage that this approach could potentially be valuable for the synthesis of mixed metal oxide catalysts, where the special properties shown here could be important for improving the activity at low temperature, enhancing stability against sintering.

## References

[cit1] Manahan S. E., Environmental Chemistry, Lewis Publishers, Boca Raton, 7th edn, 2000.

[cit2] Cho B. K. (1993). J. Catal..

[cit3] Mantri D., Aghalayam P. (2007). Catal. Today.

[cit4] Wang X., Liu D.-P., Song S.-Y., Zhang H.-J. (2013). J. Am. Chem. Soc..

[cit5] Li X.-Y., Wang X., Liu D.-P., Song S.-Y., Zhang H.-J. (2014). Chem. Commun..

[cit6] Qi J., Chen J., Li G.-D., Li S.-X., Gao Y., Tang Z.-Y. (2012). Energy Environ. Sci..

[cit7] Bao H.-Z., Zhang Z.-H., Hua Q., Huang W.-X. (2014). Langmuir.

[cit8] Li G. D., Tang Z. Y. (2014). Nanoscale.

[cit9] Chen J., Qi J., Li G. D., Zheng F. Y., Li S. X., Tang Z. Y. (2013). Chem.–Asian J..

[cit10] Chen J., Wang D. W., Qi J., Li G. D., Zheng F. Y., Li S. X., Zhao H. J., Tang Z. Y. (2015). Small.

[cit11] Liu B.-C., Wang Q., Yu S.-L., Zhao T., Han J.-X., Jing P., Hu W.-T., Liu L.-X., Zhang J., Sun L.-D., Yan C.-H. (2013). Nanoscale.

[cit12] Liu B.-C., Yu S.-L., Wang Q., Hu W.-T., Jing P., Liu Y., Jia W.-J., Liu Y.-X., Liu L.-X., Zhang J. (2013). Chem. Commun..

[cit13] Zhang J., Li L.-P., Huang X.-S., Li G.-S. (2012). J. Mater. Chem..

[cit14] Li T. Y., Xiang G. L., Zhuang J., Wang X. (2011). Chem. Commun..

[cit15] Sun C.-W., Li H., Chen L.-Q. (2012). Energy Environ. Sci..

[cit16] Bernal S., Calvino J. J., Cauqui M. A., Gatica J. M., Larese C., PeÂrezOmil J. A., Pintado J. M. (1999). Catal. Today.

[cit17] Singhania N., Anumol E. A., Ravishankar N., Madras G. (2013). Dalton Trans..

[cit18] Guo Z., Liu B., Zhang Q.-H., Deng W.-P., Wang Y., Yang Y.-H. (2014). Chem. Soc. Rev..

[cit19] Ilieva L., Pantaleo G., Nedyalkova R., Sobczak J. W., Lisowski W., Kantcheva M., Venezia A. M., Andreeva D. (2009). Appl. Catal., B.

[cit20] Tang C.-J., Sun J.-F., Yao X.-J., Cao Y., Liu L.-C., Ge C.-Y., Gao F., Dong L. (2014). Appl. Catal., B.

[cit21] Hu Y.-H., Dong L., Wang J., Ding W.-P., Chen Y. (2000). J. Mol. Catal. A: Chem..

[cit22] Archana P., Thomas E. R., Victor R., Zhu Z.-H. (2011). Catal. Today.

[cit23] Rolison D. R. (2003). Science.

[cit24] Seayad A. M., Antonelli D. M. (2004). Adv. Mater..

[cit25] Jin R. X., Yang Y., Xing Y., Chen L., Song S. Y., Jin R. C. (2014). ACS Nano.

[cit26] Zhang G.-Q., Le Y., Wu H.-B., Hoster H. E., Lou X.-W. (2012). Adv. Mater..

[cit27] Piao Y., Kim J., Bin Na H., Kim D., Baek J. S., Ko M. K., Lee J. H., Shokouhimehr M., Hyeon T. (2008). Nat. Mater..

[cit28] Li W., Deng Y.-H., Wu Z.-X., Qian X.-F., Yang J.-P., Wang Y., Gu D., Zhang F., Tu B., Zhao D.-Y. (2011). J. Am. Chem. Soc..

[cit29] Hu J., Chen M., Fang X.-S., Wu L.-W. (2011). Chem. Soc. Rev..

[cit30] Lai X. Y., Halpert J. E., Wang D. (2012). Energy Environ. Sci..

[cit31] Liu J., Qiao S.-Z., Chen J.-S., Lou X.-W., Xing X.-R., Lu G.-Q. (2011). Chem. Commun..

[cit32] Lou X.-W., Archer L. A., Yang Z.-C. (2008). Adv. Mater..

[cit33] Hu M., Chen J.-Y., Li Z.-Y., Au L., Hartland G. V., Li X.-D., Marquez M., Xia Y.-N. (2006). Chem. Soc. Rev..

[cit34] Glotzer S. C., Solomon M. J. (2007). Nat. Mater..

[cit35] Yoon M., Srirambalaji R., Kim K. (2012). Chem. Rev..

[cit36] Dhakshinamoorthy A., Garcia H. (2012). Chem. Soc. Rev..

[cit37] Li J.-R., Sculley J., Zhou H.-C. (2012). Chem. Rev..

[cit38] Zhao S. L., Yin H. J., Du L., He L. C., Zhao K., Chang L., Yin G. P., Zhao H. J., Liu S. Q., Tang Z. Y. (2014). ACS Nano.

[cit39] Zhang Z. C., Chen Y. F., He S., Zhang J. C., Xu X. B., Yang Y., Nosheen F., Saleem F., He W., Wang X. (2014). Angew. Chem., Int. Ed..

[cit40] Zhang Z. C., Chen Y. F., Xu X. B., Zhang J. C., Xiang G. L., He W., Wang X. (2014). Angew. Chem., Int. Ed..

[cit41] Rieter W. J., Pott K. M., Taylor K. M.
L., Lin W. (2008). J. Am. Chem. Soc..

[cit42] Hermes S., Witte T., Hikov T., Zacher D., Bahnmüller S., Langstein G., Hube K., Fischer R. A. (2007). J. Am. Chem. Soc..

[cit43] Guo H.-L., Zhu Y.-Z., Qiu S.-L., Lercher J. A., Zhang H.-J. (2010). Adv. Mater..

[cit44] Kim T. K., Lee K. J., Cheon J. Y., Lee J. H., Joo S. H., Moon H. R. (2013). J. Am. Chem. Soc..

[cit45] Song S.-Y., Ma J.-F., Yang J., Cao M.-H., Li K.-C. (2005). Inorg. Chem..

[cit46] Guo H. L., Zhu Y. Z., Wang S., Su S. Q., Zhou L., Zhang H. J. (2012). Chem. Mater..

[cit47] Yang W. T., Feng J., Zhang H. J. (2012). J. Mater. Chem..

[cit48] Liu K., You H.-P., Jia G., Zheng Y.-H., Song Y.-H., Yang M., Huang Y.-J., Zhang H.-J. (2009). Cryst. Growth Des..

[cit49] Liu K., You H.-P., Zheng Y.-H., Jia G., Huang Y.-J., Yang M., Song Y.-H., Zhang L.-H., Zhang H.-J. (2010). Cryst. Growth Des..

[cit50] Liu K., You H.-P., Jia G., Zheng Y.-H., Huang Y.-J., Song Y.-H., Yang M., Zhang L.-H., Zhang H.-J. (2010). Cryst. Growth Des..

[cit51] Liu K., You H.-P., Zheng Y.-H., Jia G., Zhang L.-H., Huang Y.-J., Yang M., Song Y.-H., Zhang H.-J. (2009). CrystEngComm.

[cit52] Yang Z., Wang Q., Wei S. (2011). Phys. Chem. Chem. Phys..

[cit53] Yang Z., He B., Lu Z., Hermansson K. (2010). J. Phys. Chem. C.

[cit54] Francisco M. S. P., Mastelaro V. R., Nascente P. A. P., Florentino A. O. (2001). J. Phys. Chem. B.

