# Supplementary material for: Copper doped ceria porous nanostructures towards a highly efficient bifunctional catalyst for carbon monoxide and nitric oxide elimination
Source: Chem Sci. 2015 Feb 10;6(4):2495–500. doi: 10.1039/c5sc00129c (PMC5489022; doi:10.1039/c5sc00129c)
Supplement: Supplementary file 1 [file SC-006-C5SC00129C-s001.pdf]

Electronic Supplementary Material (ESI) for Chemical Science.
This journal is © The Royal Society of Chemistry 2015

Copper Doped Ceria Porous Nanostructures towards Highly Efficient Bifunctional Catalyst for Carbon Monoxide and Nitric Oxides Elimination

Shanlong Li, Nengli Wang, Yonghai Yue, Guang-sheng Wang, Zhao Zu and Yu Zhang

Experimental Section

Materials. The initial chemicals, including $Ce(NO_3)_3 \cdot 6H_2O$ (Alfa Aescar Chemical Co.), $Cu(NO_3)_2 \cdot 3H_2O$ (Alfa Aescar Chemical Co.), benzene-1,3,5-tricarboxylic acid (BTC) (98%, Alfa Aescar Chemical Co.), terephthalic acid (BDC) (98%, Alfa Aescar Chemical Co.) 4,4'-Biphenyldicarboxylic acid (BPDC) (98%, Alfa Aescar Chemical Co.) and ethanol (A. R., Beijing Fine Chemical Company, China), were used without further purification.

Preparation.

CeO_2 nanorods: In a typical procedure, the reaction of $Ce(NO_3)_3 \cdot 6H_2O$ (1 mmol) and BTC (1 mmol) in a 1:1 stoichiometry in ethanol-water solution (50 mL, v/v = 1:1) was carried out under vigorous stirring at room temperature. The reaction mixture was then refluxed at 90 °C under constant stirring for 2 h, and a large amount of white precipitate occurred. At last, the precipitate was collected by centrifugation, washed several times with ethanol and water, and dried at 60 °C for 24 h in atmosphere. In addition, the conversion of $Ce(BTC) \cdot 6H_2O$ into CeO_2 was carried out in an oven at 600 °C for 3 h in air.

CeO_2 nanobundles: the procedure for the synthesis of CeO_2 nanobundles is similar with the procedure for the synthesis of CeO_2 nanorods. The only different is reducing the concentrations of reactant (1 mL of 0.5 M $Ce(NO_3)_3$ aqueous solution was added into BTC (0.5 mmol) water-ethanol solution (40 mL, v/v = 1:1)).

Cu^{2+}-doped CeO_2 nanorods and nanobundles: Cu^{2+}-doped CeO_2 nanorods and nanobundles were prepared by the same treatment as the undoped sample except that 0.1 mmol $Cu(NO_3)_2 \cdot 3H_2O$ and 0.9 mmol $Ce(NO_3)_3 \cdot 6H_2O$ were used instead of 1 mmol $Ce(NO_3)_3 \cdot 6H_2O$.

The procedure for the synthesis of other Cu^{2+}-doped CeO_2 nanostructures obtain from Ce(Cu)-BDC and Ce(Cu)-BPDC is similar with the procedure for the synthesis of Cu^{2+}-doped CeO_2 nanorods.

Characterization.

Powder X-ray diffraction (XRD) patterns were performed on a D8 Focus (Bruker) diffractometer (continuous, 40 kV, 40 mA, increment: 0.02 degree). Thermogravimetric analysis and differential thermal analysis (TGADTA) data were recorded with a thermal analysis instrument (SDT2960, TA Instruments, New Castle, DE) at the heating rate of 10 °C mim^{-1} in an air flow of 100 mL min^{-1}. The morphology and composition of the samples were inspected using a field emission scanning electron microscope (FE-SEM, S-4800, Hitachi) equipped with an energy dispersive X-ray spectrum (EDX, JEOL JXA-840). Transmission electron microscopy (TEM) images were obtained using a JEOL 2010 transmission electron microscope operating at 200 kV. The X-ray diffraction patterns of the products were collected on a Rigaku-D/max 2500 V X-ray diffractometer with Cu-Kα radiation (λ = 1.5418 Å), with an operation voltage and current maintained at 40 kV and 40 mA. Transmission electron microscopic (TEM) images were obtained with a TECNAI G2 high-resolution transmission electron microscope operating at 200 kV. XPS measurement was performed on an ESCALAB-MKII 250 photoelectron spectrometer (VG Co.) with Al Kα X-ray radiation as the X-ray source for excitation.

Catalytic test: 8 mg catalyst was mixed with 20 mg SiO_2. The mixture was put in a stainless steel reaction tube. The experiment was carried out under a flow of reactant gas mixture (1 % CO, 20 % O2, balance N2) at a rate of 30 mL/min. The composition of the gas was monitored on-line by gas chromatography (GC 9800).

Micromeritics ASAP2020 surface area analyzer was used to measure gas adsorption.

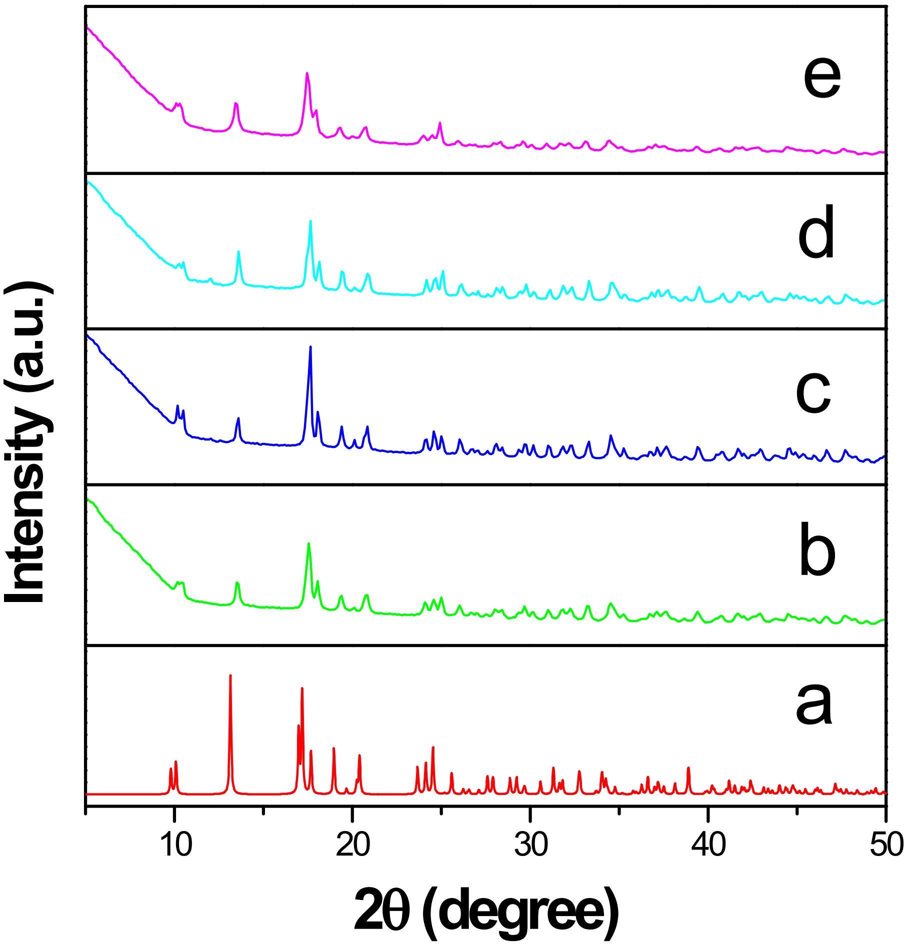

Fig. S1. The XRD patterns of $Ce(BTC)(H_2O)_6$ and Cu^{2+} doped (10% content with Ce) $Ce(BTC)(H_2O)_6$ nanocrystals (a: simulated $Ce(BTC)(H_2O)_6$; b: $Ce(BTC)(H_2O)_6$ nanorods; c: $Ce(BTC)(H_2O)_6$ nanobundles; d: $CeCu(BTC)(H_2O)_6$ nanorods; e: $CeCu(BTC)(H_2O)_6$ nanobundles).

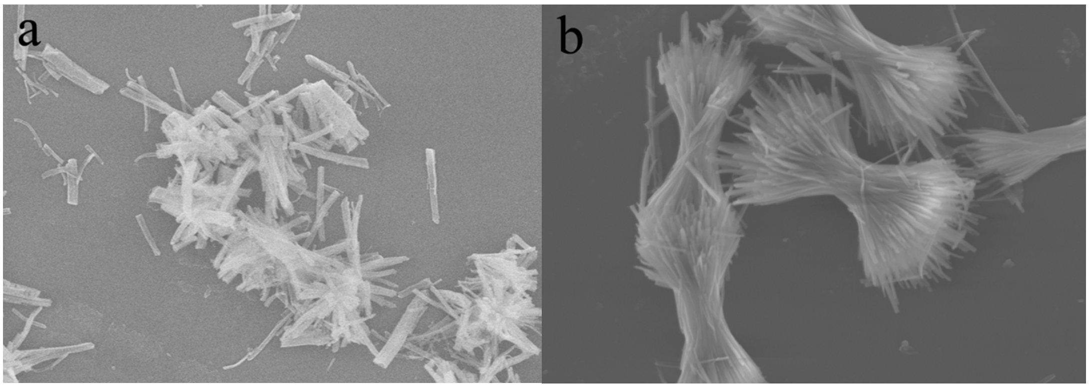

Fig. S2. The morphology of Ce-MOF nanorods (a) and nanobundles (b).

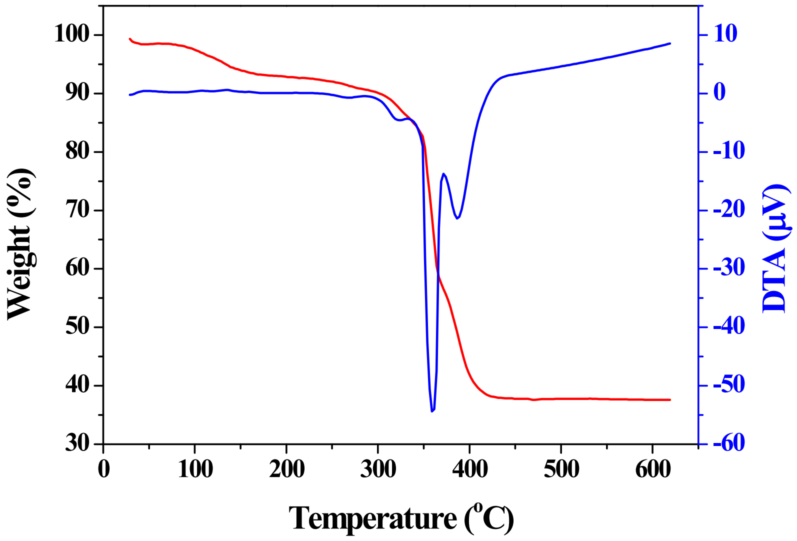

Fig. S3. TG-DTA analysis of CeCu-BTC nanocrystals.

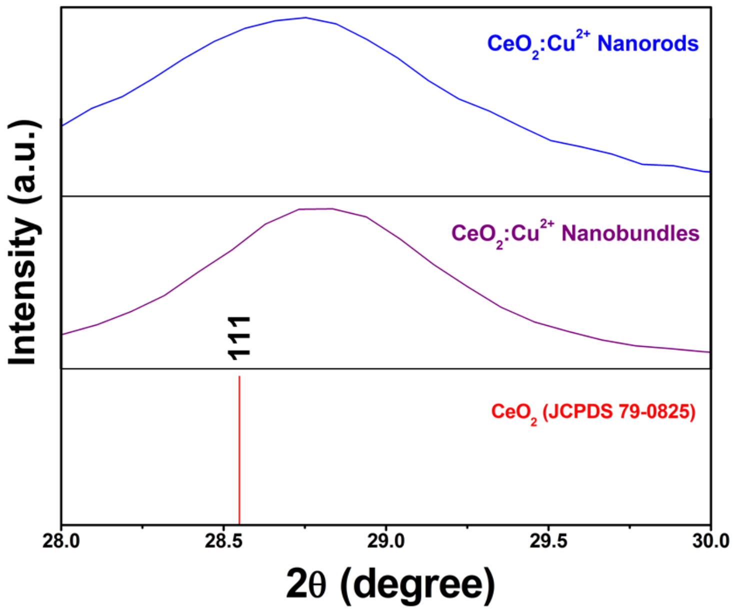

Fig. S4. XRD patterns of Cu^{2+} doped samples.

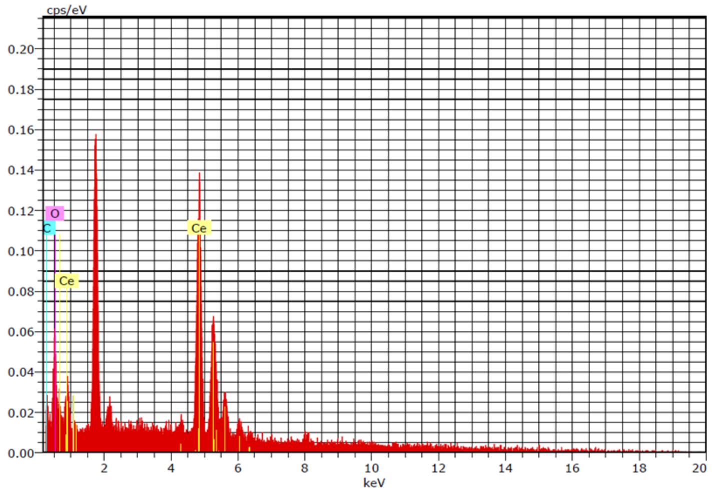

Fig. S5. The representative EDX patterns recorded from the $CeO_2:Cu^{2+}$.

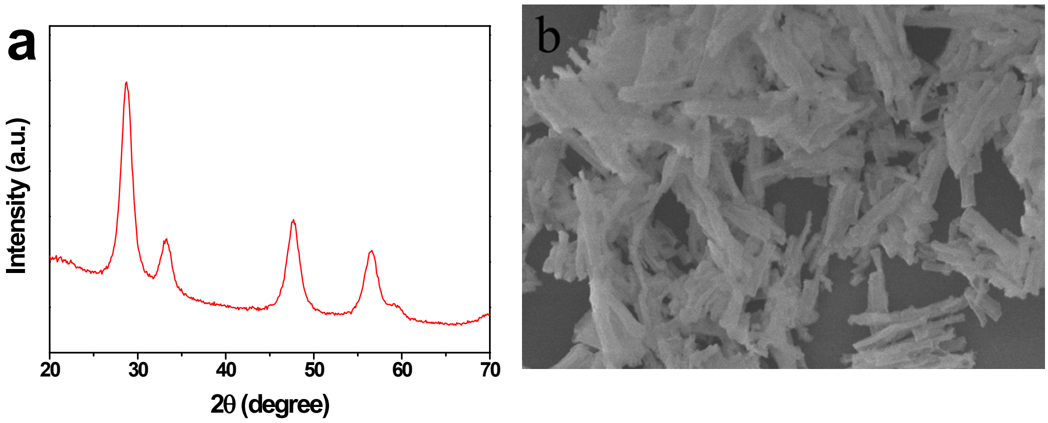

Fig. S6. The XRD pattern (a) and SEM image (b) of the nanorods after catalytic reaction.

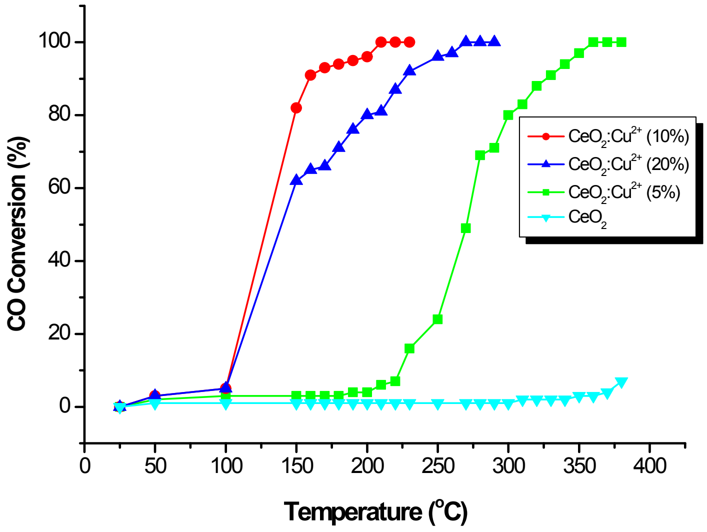

Fig. S7. The CO catalytic activity of CeO_2:Cu^{2+} porous nanomaterials with different Cu^{2+} content.

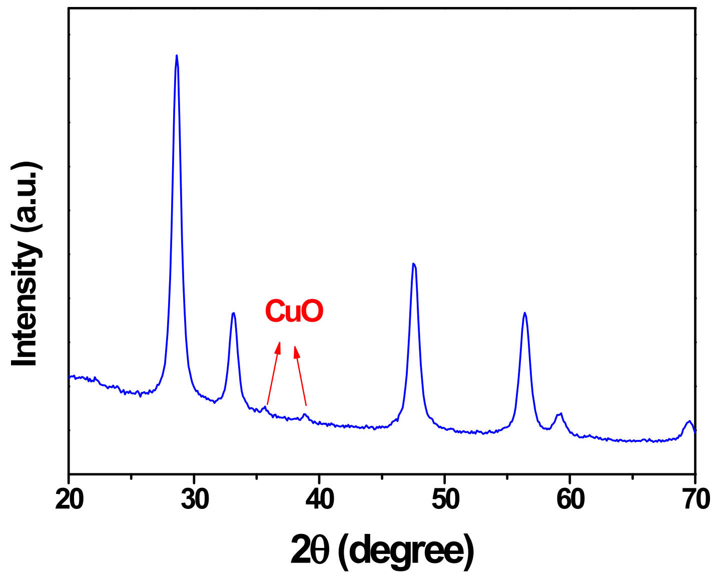

Fig. S8. The XRD pattern of CeO_2:Cu^{2+} nanocrystals with 20% Cu^{2+} content.

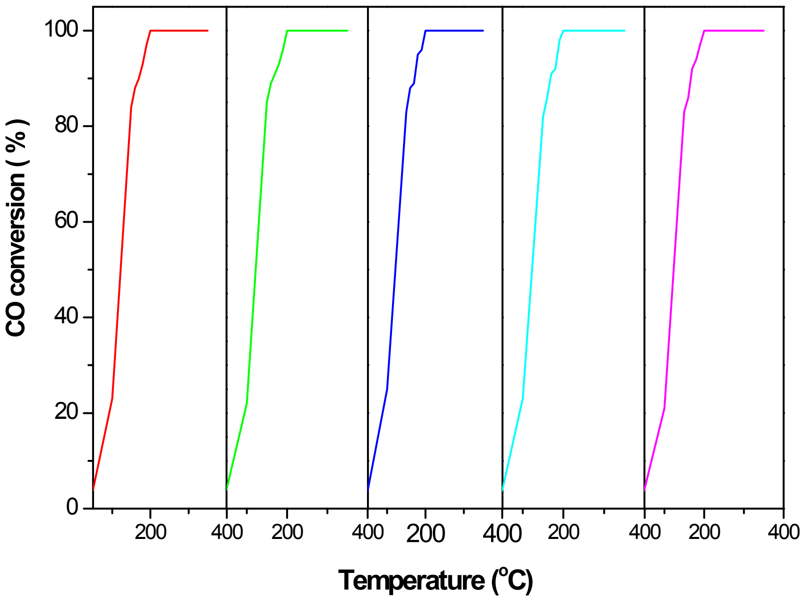

Fig. S9. Catalytic cycles of CeO_2:Cu^{2+} nanorods.

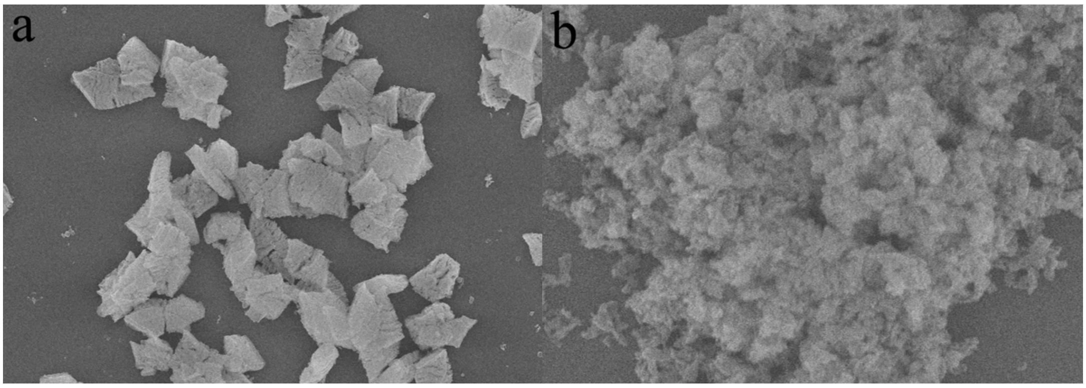

Fig. S10. The morphology of $CeO_2:Cu^{2+}$ nanocrystals obtained from CeCu-BDC (a) and CeCu-BPDC (b).
